# State of the art of rare disease activities around the world: overview of the non-European landscape

**DOI:** 10.1186/1750-1172-7-S2-A2

**Published:** 2012-11-22

**Authors:** Durhane Wong-Rieger

**Affiliations:** 1 Canadian Organization for Rare Disorders, Toronto, Ontario, Canada M5S 1S4

## 

With support from patient associations, political frameworks for rare diseases have been established throughout the world albeit with varying definitions for rare diseases.

In the USA, the National Organization for Rare Disorders was instrumental in passing the 1983 Orphan Drug Act and the 2002 Rare Disease Act, which includes medical devices and dietary products as orphan products. In 2011, the House passed bills supporting research for undiagnosed diseases and preserving regulatory fee exceptions for orphan drugs.

In 2012, the Canadian government awarded five-year rare disease research grants. With advocacy from the Canadian Organization for Rare Disorders, Health Canada concluded consultations on an orphan drug regulatory framework. Several provinces have implemented orphan drug access programs and expanded newborn screening.

In Argentina, the Geiser Foundation led advocacy resulting in the 2011 Rare Disease Law, obliging health and social systems to provide assistance. A central committee, including patients, will coordinate activities like neonatal screening and patient registries. In 2010, Colombia passed the Orphan Disease Law and hosted the 2^nd^ National Forum of Orphan Diseases. In 2011, Peru passed legislation promoting treatment and a national strategy including diagnosis, surveillance, prevention, care, and rehabilitation.

Through the 1972 Medical Care Program for Specific Diseases, Japan provides medical cost subsidy to patients affected by “56 rare and intractable diseases.” The 1993 Orphan Drug Law supports research and development. In 2008, Supporting Organizations for Patients with Rare Diseases was formed.

Since 1991, Singapore’s Orphan Drugs Policy allows patients with life-threatening and severely debilitating diseases with no other treatment options to access approved drugs prescribed by their practitioner.

The Taiwan Foundation for Rare Disorders helped secure the Rare Disease and Orphan Drugs Act in 2000. Diseases affecting fewer than 1 in 10,000 that are officially recognized are eligible for medical coverage. In Korea, the Orphan Drug Centre supplies medicines for diseases affecting fewer than 1 in 20,000. The Genetics and Rare Disease Centre supports national reference centres and research.

In China, in 2011, medical professionals called for legislation to support healthcare, research, orphan drug development, and epidemiological studies for diseases affecting fewer than 1 in 10,000.

Australia’s 1987 Orphan Drugs Policy makes available rare disease drugs, based on US regulatory information. In 2010, consultation for a national strategy was posted online. In 2012, Rare Voices Australia was formed.

